# High-Performance Limiting Current Oxygen Sensor Comprised of Highly Active La_0.75_Sr_0.25_Cr_0.5_Mn_0.5_O_3_ Electrode

**DOI:** 10.3390/s18072155

**Published:** 2018-07-04

**Authors:** Jie Zou, Qian Lin, Chu Cheng, Xin Zhang, Qinghui Jin, Han Jin, Jinxia Wang, Jiawen Jian

**Affiliations:** 1 Environmental Monitor & Sensing Technology Laboratory, School of Electrical Engineering and Computer Science, Ningbo University, Ningbo 315211, China; zoujie@nbu.edu.cn (J.Z.); linqianl7@163.com (Q.L.); chengchuu7@163.com (C.C.); zhangxin1@nbu.edu.cn (X.Z.); Jinqinghui@nbu.edu.cn (Q.J.); 2 State Key Laboratory of Transducer Technology, Chinese Academy of Sciences, Shanghai 200050, China; 3 Ningbo Materials Science and Technology Institute, Chinese Academy of Sciences, Ningbo 315201, China; 4 School of Electronic and Information Engineering, Ningbo University of Technology, Ningbo 315211, China

**Keywords:** limiting current oxygen sensor, zirconia-based, perovskite crystal phase

## Abstract

Zirconia-based limiting current oxygen sensor gains considerable attention, due to its high-performance in improving the combustion efficiency of fossil fuels and reducing the emission of exhaust gases. Nevertheless, the Pt electrode is frequently used in the oxygen sensor, therefore, it restrains the broader application due to the high cost. Quite recently, La_0.75_Sr_0.25_Cr_0.5_Mn_0.5_O_3_ (LSCM) has been reported to be highly active to catalyze oxygen reduction. Herein, with the intention of replacing the frequently used Pt, we studied the practicability of adapting the LSCM to zirconia-based limiting current oxygen sensor. Through comparing the electrocatalytic activity of LSCM and Pt, it is confirmed that LSCM gave analogous oxygen reactivity with that of the Pt. Then, limiting the current oxygen sensors comprised of LSCM or Pt are fabricated and their sensing behavior to oxygen in the range of 2–25% is evaluated. Conclusively, quick response/recovery rate (within 7s), linear relationship, and high selectivity (against 5% CO_2_ and H_2_O) in sensing oxygen are observed for the sensors, regardless of the sensing materials (LSCM or Pt) that are used in the sensor. Particularly, identical sensing characteristics are observed for the sensors consisting of LSCM or Pt, indicating the practicability of replacing the Pt electrode by adapting the LSCM electrode to future zirconia-based oxygen sensors.

## 1. Introduction

According to the Statistical Review of World Energy, fossil fuels, i.e., coal, petroleum and natural gas, contribute about 85.5% primary energy for the world [1]. Although sustainable energy, such as solar power, is gradually coming into our world, the demand of fossil fuels still continuously grows every year owing to their high converting efficiency to energy [2]. Nevertheless, the incomplete combustion of fossil fuels significantly declines the energy conversion efficiency. Moreover, burning of fossil fuels under lean burn (air rich) and/or rich burn (air lean) conditions results in the excessive emission of exhaust gases (air pollutants). Theoretically, when air/fuel (A/F) ratio is fixed at a stoichiometric point, e.g., A/F = 14.7 for gasoline, maximum energy conversion efficiency, and minimum exhaust gases emission are expected [3]. Hence, to minimize these adverse effect derives from the incomplete combustion, the high-performance oxygen sensor is adapted to the combustion system, so that the stoichiometric point of air/fuel (A/F) ratio can be maintained [4,5].

To date, oxygen sensor applied for monitoring A/F ratio includes, but not limited to, the potentiometric, limiting current, and conductometric oxygen sensors [6,7,8,9]. Among them, limiting current oxygen sensor gains more attention due to their relative stable signal, wider concentration monitoring range, and satisfactory linear relationship between response signal and oxygen concentration [6]. Typically, the conventional limiting current oxygen sensor comprises of noble Pt electrodes, 8YSZ electrolyte and diffusion barrier. Although Pt electrode demonstrates incomparable high catalytic activity to oxygen so that giving superior sensing performance [6,8], its high price results in the increase on the cost of the oxygen sensor, and therefore, it restrains the widespread of this kind of oxygen sensor in the market. Consequently, there is the need to find counterpart alternatives to replace the Pt electrode that is frequently used in the limiting current oxygen sensor. Up to now, lots of strategies have been proposed to overcome this challenging issue. For instance, to reduce the use of Pt in the limiting current oxygen sensor, CaZr_0.7_Mn_0.3_O_3_ (M = Fe, Cr, and Co) has been explored as the dense diffusion barrier [10]. In addition, Shunsuke Akasaka et al., developed a limiting current oxygen sensor with the novel configuration and the sensor show excellent limiting current plateau at 450–550 °C [11]. Apart from the CaZrO_3_-based sensing materials, SrTiO_3_-based perovskites also gained intensity attention due to their excellent surface conductivity and phase transition when exposed to oxygen [12,13]. However, so far, as we know, there is rare report on finding alternatives to replace the using of Pt electrode in the zirconia-based limiting current oxygen sensor.

Inspired by the Solid-state Oxide Fuel Cells (SOFCs), La_0.75_Sr_0.25_Cr_0.5_Mn_0.5_O_3_ (LSCM), which works as the cathode or anode is expected to be the potential alternative for Pt replacement, since the LSCM is confirmed to be highly active for oxygen catalysis [14,15,16]. Meanwhile, LSCM can form imitate contact with YSZ no matter under oxidation or reducing atmosphere at high temperatures. Herein, in this research LSCM is used as the electrodes in zirconia-based limiting current oxygen sensor and the sensing performance of the designed sensor will be systematically studied to confirm our assumption, namely, the practicability of replacing Pt electrodes.

## 2. Materials and Methods

### 2.1. Powders Preparation

LSCM powders were synthesized by sol-gel method. The starting materials were La(NO_3_)_3_·6H_2_O (Alpha, AR), Sr(NO_3_)_2_ (Aladdin, Shanghai, China, AR), Cr(NO_3_)_3_·9H_2_O (Aladdin, AR), and MnNO_3_ solution (Aladdin, Shanghai, China, AR, 50%). EDTA (Aladdin, Shanghai, China, Ethylenediamineteraacetic acid, AR) and citric acid (Alfa Aesar, Shanghai, China, AR) are used as complexing and polymerizing agents, respectively. These raw materials and additives were dissolute in deionized water and the final solution was stirred at 80 °C until becoming gelation. The gel was pyrolized at 200 °C and the powder precursors were calcined at 1100, 1200, 1300, and 1400 °C for 4 h to form the desired structure. The purity of the compounds was checked by X-ray diffraction with Cu-Kα radiation (XRD, D8 advance, Bruker, Billerica, MA, USA).

### 2.2. Samples Fabrication

**Figure 1:**
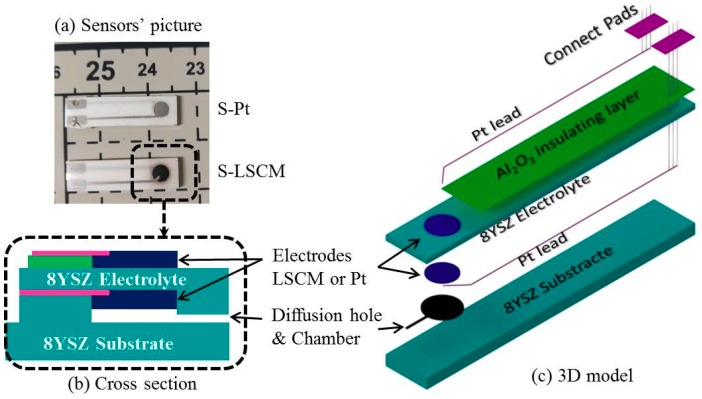
Schematic diagram of limiting current oxygen sensor.

Two types of cell with asymmetric sandwich structure were fabricated by HTCC technology to evaluate electrochemical activity of LSCM and Pt that are attached on the surface of 8YSZ electrolyte. Limiting current sensor with Pt or LSCM electrodes were also prepared by HTCC technology. Commercially available 8YSZ powders (Tosoh, Japan), Al_2_O_3_ powders (Taimei chemicals, Tokyo, Japan), Pt-7851, Pt-7850 (Sino-platinum metals, Guiyang, China), and self-synthetic LSCM powders were fabricated into electrolytes layer, insulating layer, Pt leaders, Pt electrodes, and new electrodes, respectively. Carbon (Cancarb, Medicine Hat, AB, Canada) powder was used to form diffusion hole and diffusion chamber. Firstly, the 8YSZ tape was home-fabricated by tape casting, the detail process has been reported in previous paper [17] and supplemented. In this paper, the blade height is 600 μm, after drying, the thickness of green tape was about 200 μm. Secondly, LSCM, carbon, and Al_2_O_3_ printing inks powders were prepared by mix the powder with α-terpineol, respectively. Thirdly, the 8YSZ cells with symmetric sandwich structure were formed by printed Pt-7850 or LSCM inks, as electrodes on the surface of 8YSZ green tape, respectively. Fourthly, the sensors are showed in Figure 1a. Fabricating by following steps:

(1) The Al_2_O_3_ ink was screen printed on the right top of an 8YSZ green tape as insulting layer.
(2) The electrode inks (LSCM or Pt-7850) were screen-printing on the top and button of the green tape’s right side as electrode.
(3) Pt-7851 was printed as Pt leader and connect pads, as shown in Figure 1c.
(4) The carbon ink was printed on the button of them, which will be burned out during sintering. A small hole will be produced and it will be used as diffusion barrier. At the same time, a chamber is fabricated around the inner electrode.
(5) Six layers of 8YSZ tape were laminated on the bottom as substrate to get enough mechanical strength. The final cross section of sensor is shown in Figure 1b.
(6) The cells and sensors were both isotactic pressed at 80 °C and 60 Mpa in water, followed by sintering at 1400 °C for 4 h.

Finally, the cells with LSCM and Pt electrodes were marked as C-LSCM and C-Pt; the sensors with LSCM and Pt electrodes were marked as S-Pt and S-LSCM, respectively. The microstructures of cells and sensors were examined by and scanning electron microscopy (SEM, SU-70, Hitachi, Tokyo, Japan).

### 2.3. Evaluation of the Electrochemical Activity

To evaluate the electrocatalytic activity of 8YSZ cells to oxygen, electrochemical impedance spectroscopy (EIS) was applied by AC impendence meter (HIOKI 3522-50, Ueda, Japan). The AC impedance spectra were obtained at 380, 420, 460, 500, 540, 580, and 620 °C under an excitation of 20 mV over the frequency range from 1 mHz to 100 kHz in 5% O_2_ containing dry air. The impedance spectra were fitted by Zview3.2 program by using the equivalent circuit R_s_(R_1_Q_1_) (R_2_Q_2_), where R_s_ is the ohmic resistance and Q is the constant phase element [18,19]. The electrochemical activity was evaluated from impedance spectra by the difference between the high-frequency and low-frequency intercepts with the real axis.

### 2.4. Evaluation of the Sensing Performance

To evaluate the sensing performance of the sensors at 380–620 °C, gas sensing was performed in a conventional gas-flow apparatus. The oxygen concentration and flow rate were fixed at 1, 2, 5, 10, 15, 20, 25%, 30%, and 200 SCCM, respectively. Linear sweep voltammetry (LSV) were carried out to evaluate the limiting current platform of sensors by 98B2 (Lanlike, Tianjin, China) electrochemical workstation. The scanning voltage was changed from 0–1500 mV with a sweep speed fixed at 0.2 mV/s. The response curves were tested with a 0.5 V pumping voltage was applied by negative and positive poles are connected to inner and external electrodes, respectively. Selectivity was evaluated between O_2_, CO_2_, and H_2_O with the oxygen concentration was fixed at 5% and H_2_O was induced by bubbling method.

## 3. Results and Discussion

With the intention of replacing the frequently used Pt electrode with the LSCM, a zirconia-based limiting current oxygen sensor comprises of LSCM electrodes will be fabricated. Then, sensing characteristics of the sensor under different oxygen concentration will be evaluated. Since, LSCM is believed to be highly activity to oxygen, it is expected that the zirconia-based limiting current oxygen sensor comprises of LSCM electrodes should demonstrate analogous sensing performance with that of the sensor using Pt electrodes.

**Figure 2:**
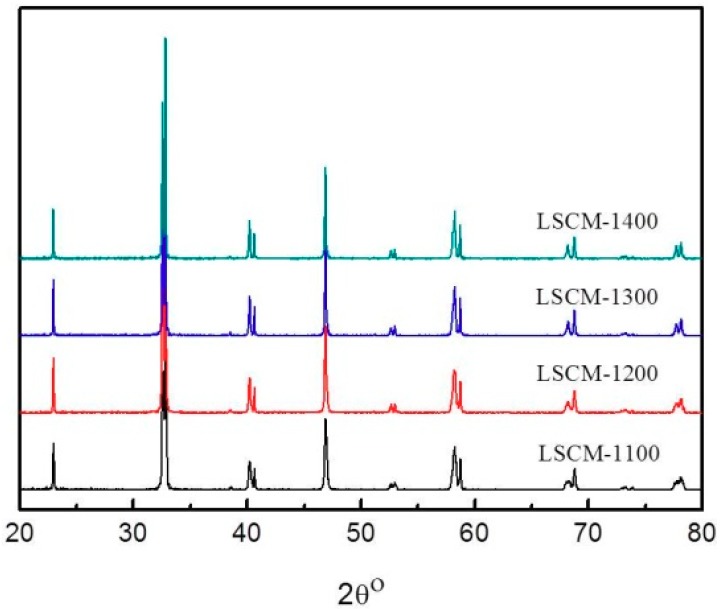
X-ray diffraction (XRD) patterns of the La_0.75_Sr_0.25_Cr_0.5_Mn_0.5_O_3_ (LSCM) powders calcined at the temperature of 1100–1400 °C.

**Figure 3:**
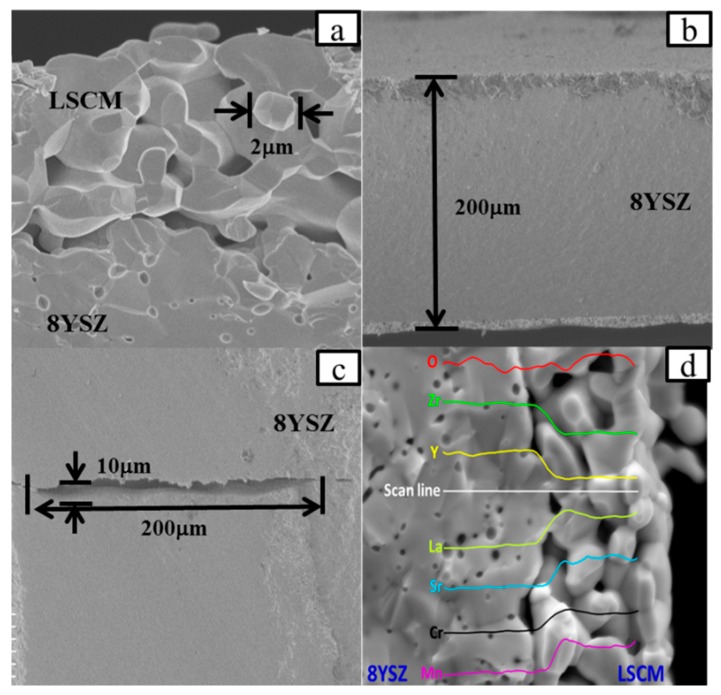
Scanning electron microscopy (SEM) images of (**a**) LSCM powder calcined at 1100 °C; (**b**) Micro-structure of LSCM electrode after 1400 °C co-firing with 8YSZ; (**c**) Diffusion barrier of sensor; and, (**d**) Energy Disperse Spectroscopy (EDS) image at line scan mode.

Based on the above-mentioned assumption, initially the influence of calcination temperature on the crystallographic phase of LSCM is studied to optimize the fabrication and operational conditions of the sensor. Figure 2 shows the XRD patterns of the LSCM that was calcined at the temperature of 1100–1400 °C. LSCM with pure perovskite crystal phase can be found and even be stable after thermal treated at 1400 °C. Typically, LSCM can only form the imitate contact with dense 8YSZ at the temperature above 1400 °C, due to the limitation of HTCC technology [20]. Since LSCM with the perovskite phase can be remained at such a high temperature, the fabrication temperature of the sensor in the following research is fixed at 1400 °C. As can be seen in Figure 3, after thermal treated at 1400 °C, the diameter of the particle size for the LSCM obtained via sol-gel way is about 2 μm (Figure 3a), and such porous LSCM is beneficial for oxygen molecular arriving at the LSCM/8YSZ reaction interface (three phase boundary (TPB)). Particularly, the interface between the LSCM and 8YSZ is hardly defined, suggesting the satisfactory contact is formed (Figure 3a). Additionally, by using the screen-printing technology, the thickness of the LSCM layer and 8YSZ is 20 μm and 200 μm, respectively (Figure 3b). A perfect diffusion barrier with the dimensions of 3000, 200 and 10 μm (length × width × height is obtained and its SEM image is shown in Figure 3c). Since, it is reported that LSCM can form solid solution with YSZ at high calcination temperature, thus, EDX is taken on the sample to confirm the existence of LSCM-YSZ solid solution. Figure 3d gives the EDX image under line scan mode and confirms that, after thermal treatment, LSCM did not form any solid solution with 8YSZ.

**Figure 4:**
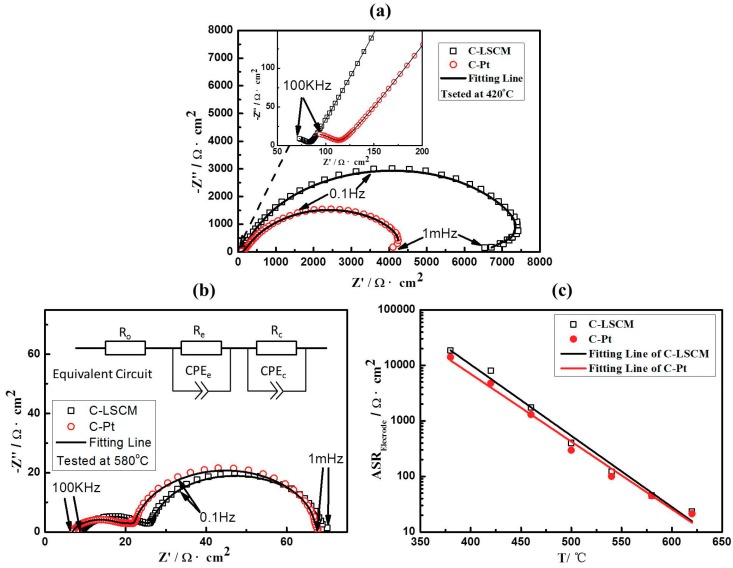
EIS of C-LSCM and C-Pt tested at (**a**) 420 °C and (**b**) 580 °C; and, (**c**) The relationship between area resistance and operating temperature of C-LSCM and C-Pt.

The LSCM can replace the Pt as the electrode in the limiting current oxygen sensor only if its catalytic activity to oxygen is equivalent to that of the Pt. Consequently, the reaction activity of the LSCM is measured at the operational temperature of 380–620 °C (appearing in the form of Nyquist curves), and compared with that of the Pt at the same operational conditions (shown in Figure 4 and Figure S1 of Supplementary Materials). In brevity, with the increase in the operational temperature, the nyquist curves of LSCM and Pt tends to be identical. For instance, at the temperature of 420 °C (Figure 4a), apparent difference for the resistance (at 1 mHz) that estimated at the cross-section point of x-axis is found for the Nyquist curves of LSCM and Pt, although the value of the resistance is at the same order of magnitudes. In contrast, when the operational temperature is above 580 °C (Figure 4b), identical Nyquist curves are observed. The value of the resistance (at 1 mHz) versus the temperature is plotted and shown in Figure 4c. Interestingly, it is found that the variation tendency of the resistance (at 1 mHz) derived from LSCM matched well with the change tendency of Pt. Since the Nyquist curves recorded at low frequency represents the interfacial catalytic activity of the materials, it is reasonable to deduce that the electrocatalytic activity to oxygen for LSCM is very close to that of the Pt. This speculation is further studied by evaluating the sensing characteristics of the limiting current oxygen sensor while using LSCM or Pt electrodes.

**Figure 5:**
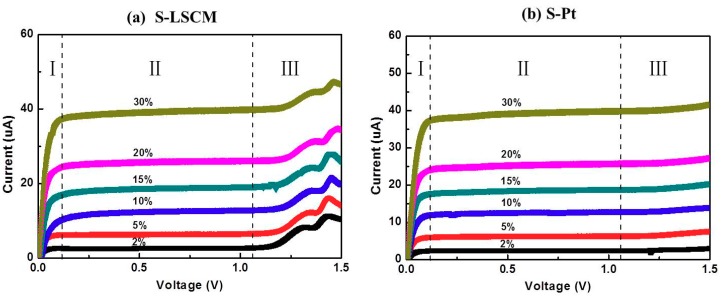
Current-voltage characteristic curves of (**a**) S-LSCM and (**b**) S-Pt, measured at 580 °C, with the oxygen concentration in the range of 2–30%.

**Figure 6:**
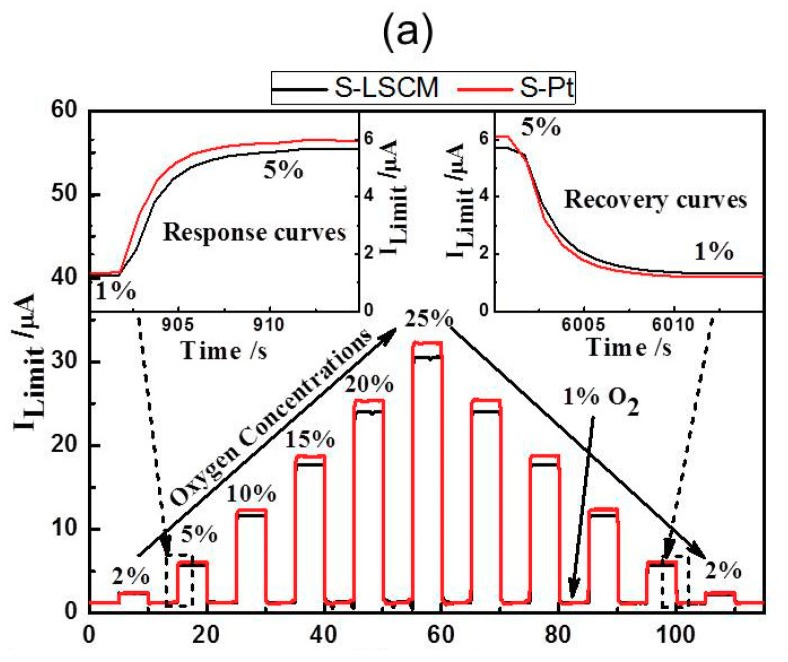

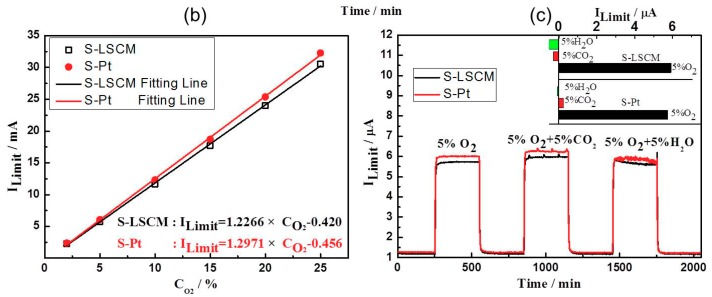
(**a**) Response transient and (**b**) Dependency of the response signal on the oxygen concentration in the range of 2–25% for the sensors comprised of LSCM or Pt electrode; (**c**) Selectivity of the S-LSCM and S-Pt against 5% CO_2_ and H_2_O, at the operational temperature of 580 °C.

Figure 5 gives the current-voltage (I-V) characteristic curves of the S-LSCM and S-Pt at different oxygen concentrations (measured at 580 °C), with applying the bias voltage in the range of 0–1.5 V. Generally, when the applied bias voltage is below 0.1 V, the current mainly increases linearly with increasing voltage. In this case, the ionic conductivity of YSZ solid electrolyte is dominant before the limiting current occurs at the voltage of lower than 0.1 V. Then, with continuously increasing the bias voltage, limiting current plateau is observed in a wide voltage range of 0.1–1 V. Such satisfactory sensing behavior is due to the excellent electrocatalytic activity of LSCM and Pt, high ionic conductivity of 8YSZ, and desirable diffusion efficiency of the barrier. Nevertheless, the current value increases non-linearly if further increasing the bias voltage. This indicates that the optimal bias voltage for the sensor is within the range of 0.1 to 1 V. Besides, it should be additionally noted that based on the similar I-V characteristics of LSCM (Figure 5a) and Pt (Figure 5b), it can be concluded that LSCM is highly possible to replace the Pt for oxygen sensing. Accordingly, sensing performance of the sensor consisting of LSCM or Pt electrode is evaluated at 580 °C with applying the bias voltage of 0.5 V. The sensor using LSCM electrode gives close sensing behavior with that of the sensor using Pt electrode (Figure 6a and Figure S2 of Supplementary Materials). For example, limiting currents of S-LSCM and S-Pt tested at 20% oxygen are 24.029 and 24.009 μA, respectively. The relative error is within 0.08%. Meanwhile, the response signal of the sensor changes quickly with the changes in the oxygen concentration in the range of 2–25%, and it reaches the steady state in a short time (insect of Figure 6a), the 90% response/recovery time is normally within 7 s, even at the operational temperature down to 420 °C (Figure S3 of Supplementary Materials). Apart from the quick response/recovery rate, a perfect linear relationship between the response signal and oxygen concentration within the tested concentration is witnessed (Figure 6b). The correlation coefficient for the sensor using LSCM or Pt electrode reaches 0.999. For the purpose of adapting the developed limiting oxygen sensor to the real application, resistance to other interfering gases is an ultra-important parameter [21]. Thus, sensing behavior of the limiting current oxygen sensors (utilizing LSCM or Pt electrode) against 5% CO_2_ or H_2_O is investigated (shown in Figure 6c). The sensors give acceptable selectivity to oxygen since their response signal is hardly affected by the 5% CO_2_ or H_2_O. Furthermore, the identical sensing behavior for the sensor comprised of LSCM or Pt electrode confirms our speculation that LSCM indeed demonstrates high reactivity to oxygen and can replace the Pt to be used in the limiting current oxygen sensor. Table 1 summarized the performance of the reported oxygen sensor using various sensing materials [6,7,10,13,22,23]. In summary, the limiting oxygen sensor comprised of LSCM shows comparable sensing behavior with that of these mentioned counterparts, and minor hysteresis is observed for the sensor using LSCM or Pt electrode with the bias voltage that is below 0.5 V (Figure S4). The negligible hysteresis of the LSCM can be attribute to the tiny polarization at the voltage of 0–0.5 V. Regarding to the high catalytic activity of LSCM to oxygen, it can be interpreted through its high oxygen vacancy concentration. Generally, oxygen reduction over an electrode involves a series of steps, such as gas phase diffusion, oxygen dissociation, and surface diffusion of oxygen ion into the electrolyte layer in the TPB region. The oxygen dissociation and surface diffusion of oxygen ions are closely related to the surface oxygen vacancy concentration. Due to the nonstoichiometry structure of LSCM [14], it contains considerable amounts of oxygen vacancies, leading to oxygen dissociation and surface diffusion of oxygen ions easily, which is rate determining step of catalysis. oxygen dissociation and surface diffusion of oxygen ions can be easily proceeded to rate determining step (i.e.,. Accordingly, the LSCM demonstrates analogous catalytic activity to oxygen with that of Pt. Further discussion can be found elsewhere [14].

## 4. Conclusions

The practicability of adapting LSCM to the limiting current oxygen sensor is systematically studied. The LSCM synthesized via sol-gel way shows a high thermal stability, and even remained its perovskite crystal phase at 1400 °C. Through screen-printing method, zirconia-based limiting current oxygen sensors comprised of LSCM or Pt electrode are fabricated. By means of measuring the impedance spectra, the electrocatalytic activity to oxygen for the LSCM and Pt electrode is investigated and compared. Then, response behavior of the sensor using LSCM or Pt electrode to oxygen in the range of 2–25% is studied. In summary, LSCM demonstrates analogous oxygen reactivity with that of Pt. Moreover, identical sensing behavior of the sensor consisting of LSCM or Pt electrode is confirmed, and these sensors demonstrate high resistance to 5% CO_2_ and H_2_O, as well as linear relationship for oxygen sensing. These promising results indicate the fact that the LSCM can be utilized in limiting oxygen sensor to replace the frequently used Pt electrode in the future.

## Figures and Tables

**Table 1:** Compassion of the sensing behavior for the oxygen sensors using various sensing materials.

Sensing Materials	Operating Mode	Concentration Range	Operating Temperature	Reference
Pt	Amperometric/potentiometric	0–21%	Above 350 °C	[6,7]
BaFeO_3_	Impedancemetric	0.2–21%	600 °C	[23]
SrTi_0.6_Fe_0.4_O_3__−_*_δ_*	Conductometric	1–100%	400–750 °C	[13]
TiO_2_		0.05–2.5%	Below 500 °C	[22]
CaZr_0.7_Mn_0.3_O_3_	Amperometric	0–21%	650–830 °C	[10]
La_0.75_Sr_0.25_Cr_0.5_Mn_0.5_O_3_		2–25%	Above 350 °C	This research

## Supplementary Materials

The following are available online at http://www.mdpi.com/1424-8220/18/7/2155/s1. 1. Detail processes of LSCM synthesis by sol-gel method; 2. Detail processes of 8YSZ tape casting; 3. Figure S1. Comparisons of the EIS of C-LSCM and C-Pt tested from 380 to 620 °C; 4. Figure S2. Comparisons of the response behavior of S-LSCM and S-Pt tested from 380 to 620 °C; 5. Figure S3. The relationship between average 90% response/recovery time and the operating temperature for the S-LSCM and S-Pt; 6. Figure S4. Cyclic voltammetry of the S-LSCM and S-Pt within the bias voltage of 0–1.5 V.
