# Supplementary material for: High-Performance Limiting Current Oxygen Sensor Comprised of Highly Active La0.75Sr0.25Cr0.5Mn0.5O3 Electrode
Source: Sensors (Basel). 2018 Jul 4;18(7):2155. doi: 10.3390/s18072155 (PMC6069003; doi:10.3390/s18072155)
Supplement: Supplementary file 1 [file sensors-18-02155-s001.pdf]

Supporting Information for:

High-Performance Limiting Current Oxygen Sensor Comprised of Highly Active $La_{0.75}Sr_{0.25}Cr_{0.5}Mn_{0.5}O_3$ Electrode

Jie Zou [1], Qian Lin [1], Chu Cheng [1], Xin Zhang [1], Qinghui Jin [1,2], Han Jin [1,3,*], Jinxia Wang [4,*] and Jiawen Jian [1,*]

[1] Environmental Monitor & Sensing Technology Laboratory, School of Electrical Engineering and Computer Science, Ningbo University, Ningbo 315211, China; zoujie@nbu.edu.cn (J.Z.); linqianl7@163.com (Q.L.); chengchuu7@163.com (C.C.); zhangxin1@nbu.edu.cn (X.Z.); Jinqinghui@nbu.edu.cn (Q.J.)

[2] State Key Laboratory of Transducer Technology, Chinese Academy of Sciences, Shanghai 200050, China

[3] Ningbo Materials Science and Technology Institute, Chinese Academy of Sciences, Ningbo 315201, China

[4] School of Electronic and Information Engineering, Ningbo University of Technology, Ningbo 315211, China

* Correspondence: jinhan@nbu.edu.cn (H.J.); jianjiawen@nbu.edu.cn (J.W.); jianjiawen@nbu.edu.cn (J.J.); Tel.: +86-574-8760-9493 (J.W.)

Supporting Information Available: The following files are available free of charge.

1.Detail processes of LSCM synthesis by sol-gel method

2. Detail processes of 8YSZ tape casting.

3. Figure S1. Comparisons of the EIS of C-LSCM and C-Pt tested from 380 to 620°C;

4. Figure S2. Comparisons of the response behavior of S-LSCM and S-Pt tested from 380 to 620°C;

5. Figure S3. The relationships between average 90% response/recovery time and operating temperature for the S-LSCM and S-Pt.

1. LSCM synthesis process.

LSCM powders were synthesized by sol-gel method. The starting materials were $La(NO_3)_3\cdot 6H_2O$ (Alpha, AR), $Sr(NO_3)_2$ (Aladdin, AR),$Cr(NO_3)_3\cdot 9H_2O$ (Aladdin, AR) and

$MnNO_3$ solution(Aladdin, AR, 50%). EDTA (Aladdin, Ethylenediamineteraacetic acid, AR) and citric acid (Alpha, AR) are used as complexing and polymerizing agents, respectively. Ammonium hydroxide (Sinopharm, AR, 25~28%) was added to promote the dissolution of EDTA in deionized water. Then, the basic solution of EDTA and ammonia was added drop wise to an aqueous solution containing stoichiometric amounts of La, Sr, Cr and Mn. After the addition was completed, a measured amount of citric acid was added (metal nitrates: EDTA: citric acid molar ratio=1:1.5:1). These raw material and additive was dissolute in deionized water and the final solution was stirred at 80°C until gelation. The gel was pyrolized at 200oC and the powder precursors were calcined at 1100, 1200, 1300, 1400°C for 4h to remove residual organic and form the desired structure.

2. 8YSZ tape casting process.

Firstly, the 8YSZ powders were homogeneously dispersant in a planetary mill for 1h using zirconia balls, acrylic, xylene and butyl acetate as milling media, dispersant, and solvents, respectively. Then polyvinyl butyral (PVB) and polyethylene glycol (PEG) were added as binder and plasticizer, respectively. The mixture was followed by planetary mill for another 2h. The compositions of scurry for 8YSZ electrolyte are shown in Tab.1.

Tab.1 Compositions of 8YSZ electrolyte for tape casting

Composition of the tape casting slurry (Wet. %)					
Power	solvent		Dispersant	Binder	Plasticizer
8YSZ	Xylene	Butyl acetate	Acrylic	PVB	PEG
67	11	11	2	5	4

Before tape casting, the slurry was vacuum-pumped for 10 min to remove air. The 8YSZ electrolyte film was casted on a Mylar substrate at the blade height of 600μm, after drying, the thickness of green tape was about 200μm.

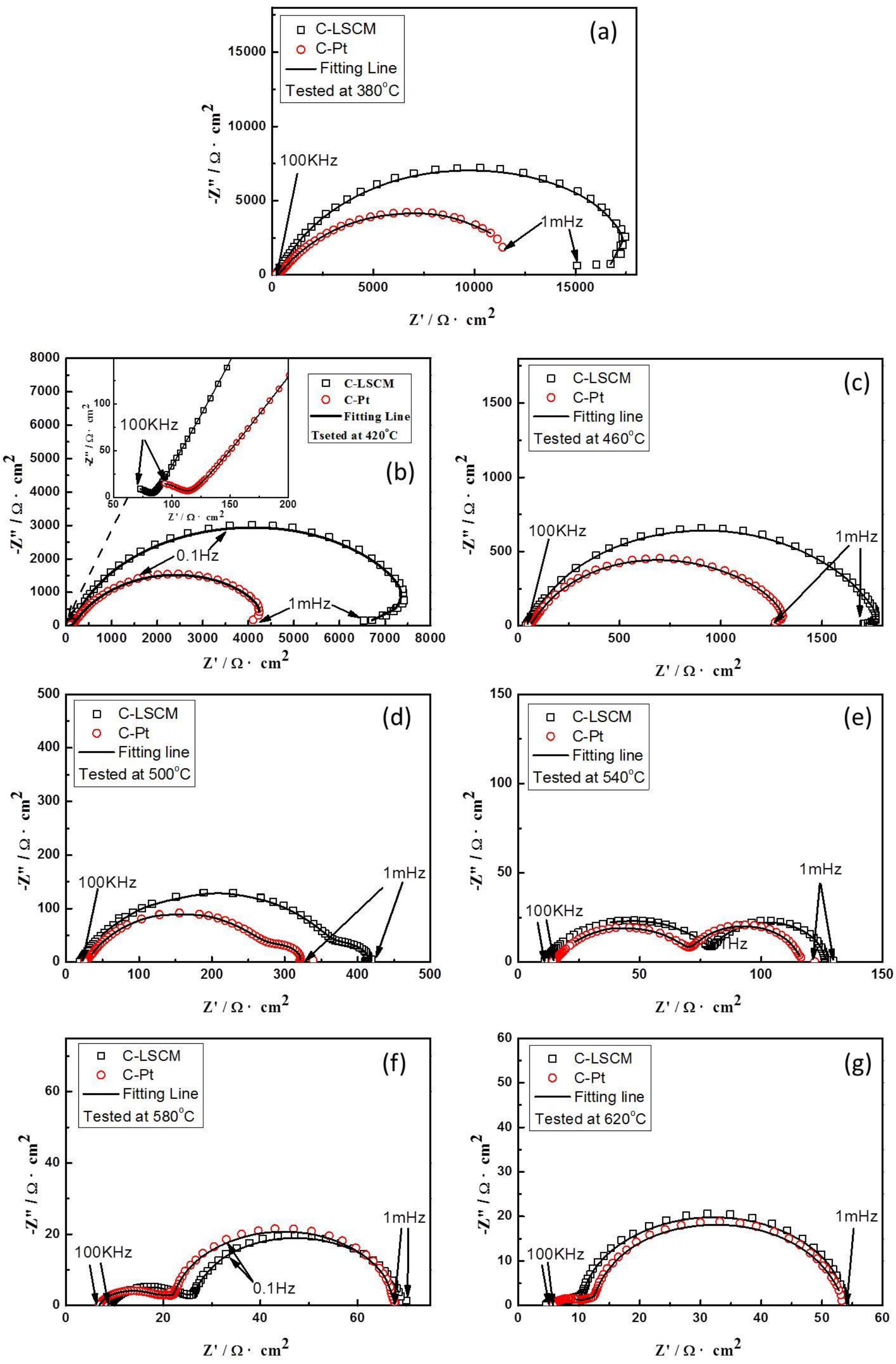

Figure S1. Comparisons of the EIS of C-LSCM and C-Pt tested from 380 to 620°C.

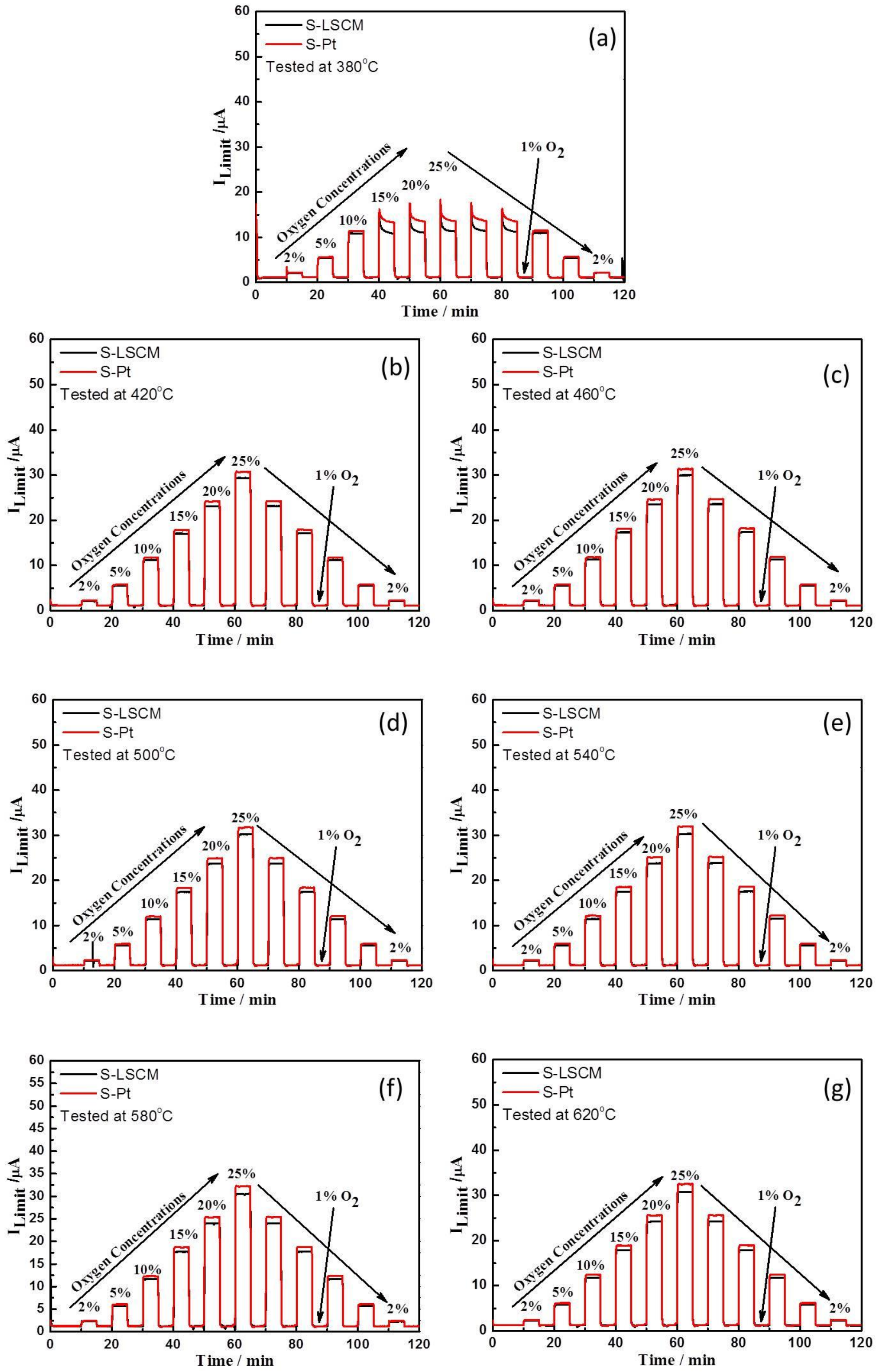

Figure S2. Comparisons of the response behavior of S-LSCM and S-Pt tested from 380 to 620°C.

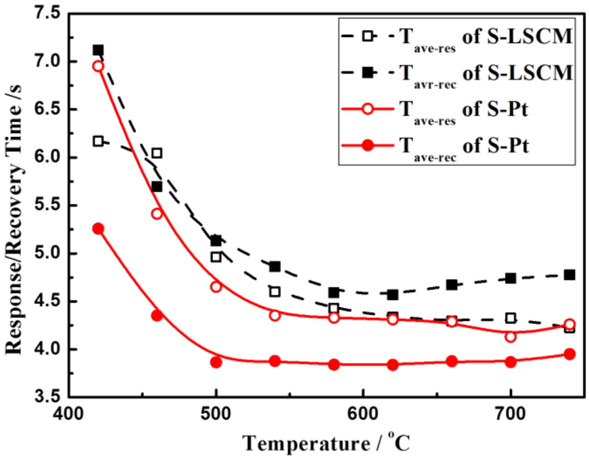

Figure S3. The relationship between average 90% response/recovery time and the operating temperature for the S-LSCM and S-Pt.

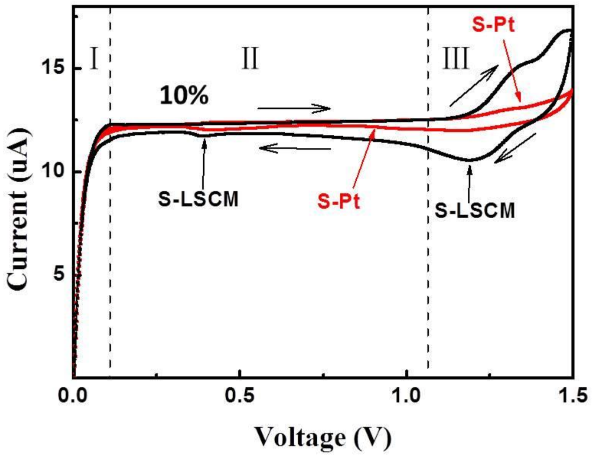

Figure S4. Cyclic voltammetry of the S-LSCM and S-Pt within the bias voltage of 0-1.5V.
